# Mapping soil organic carbon stocks in Nepal’s forests

**DOI:** 10.1038/s41598-023-34247-z

**Published:** 2023-05-19

**Authors:** Shiva Khanal, Rachael H. Nolan, Belinda E. Medlyn, Matthias M. Boer

**Affiliations:** 1 Forest Research and Training Center, Kathmandu, Nepal; 2 Hawkesbury Institute for the Environment, Western Sydney University, Sydney, Australia

**Keywords:** Ecology, Ecological modelling, Forestry

## Abstract

Comprehensive forest carbon accounting requires reliable estimation of soil organic carbon (SOC) stocks. Despite being an important carbon pool, limited information is available on SOC stocks in global forests, particularly for forests in mountainous regions, such as the Central Himalayas. The availability of consistently measured new field data enabled us to accurately estimate forest soil organic carbon (SOC) stocks in Nepal, addressing a previously existing knowledge gap. Our method involved modelling plot-based estimates of forest SOC using covariates related to climate, soil, and topographic position. Our quantile random forest model resulted in the high spatial resolution prediction of Nepal’s national forest SOC stock together with prediction uncertainties. Our spatially explicit forest SOC map showed the high SOC levels in high-elevation forests and a significant underrepresentation of these stocks in global-scale assessments. Our results offer an improved baseline on the distribution of total carbon in the forests of the Central Himalayas. The benchmark maps of predicted forest SOC and associated errors, along with our estimate of 494 million tonnes (SE = 16) of total SOC in the topsoil (0–30 cm) of forested areas in Nepal, carry important implications for understanding the spatial variability of forest SOC in mountainous regions with complex terrains.

## Introduction

Accurate and robust estimation of national soil organic carbon (SOC) stocks is crucial for reporting in the context of the United Nations Reducing Emissions from Deforestation and Forest Degradation (REDD+) program^1^ and several other initiatives^2^. Assessing carbon stocks in forest ecosystems requires understanding biomass in trees, belowground biomass, and soil carbon, as well as the fluxes. Estimating belowground biomass is more challenging and costly than aboveground biomass^3^. Several REDD+ countries have omitted soil carbon pools from emission reporting due to technical challenges in monitoring stocks^4^ and have focused mainly on aboveground biomass. Spatially explicit SOC estimates improve our understanding of the carbon cycle^5^, support monitoring of changes over time, and inform national and international climate change mitigation policies^6^. However, estimating SOC stock for an entire country is a major challenge due to the lack of reliable data in many regions and high uncertainty in existing estimates, mainly due to limited observations^7^. Additionally, assessments of carbon dioxide uptake have neglected the role of soil carbon stocks and their climate sensitivity^8^. The highly variable nature of this carbon stock, as demonstrated by contrasting estimates for different forest types across the world^9^, highlights the importance of producing reliable estimates specific to different regions.

The knowledge of the spatial distribution of soil organic carbon (SOC) in mountainous regions is currently limited, partly due to the challenges in sampling complex terrain with limited accessibility^10,11^. Extrapolating findings on the distribution and expected changes in SOC stocks from well understood regions elsewhere may not be suitable for mountainous regions in other continents, due to variations in climate, topography, and land-use history^12^. Natural and human-made disturbances such as landslides^13^, erosion^14^, fire^15^, and land-use changes^16,17^ can cause forest SOC loss. The effects of climate change, including rising air temperatures and increased decomposition rates can also increase $\hbox {CO}_{2}$ emissions^18,19^. Improving our understanding of the spatial variation of carbon stocks in mountainous regions will guide adaptation and mitigation measures for climate change impacts.

Soil formation depends on many factors and can be expressed as a function of several key controls^20^:

1 $$\begin{aligned} soils = f(parent\ material, \ climate, \ relief, \ organisms, \ humans, \ time) \end{aligned}$$

In order to make a spatially explicit prediction of soil attributes, location information is required in addition to attributes such as type of parent material, climate, relief and land-use history. Building on Eq. (1), the ‘scorpan’ model^21^ is expressed as:

2 $$\begin{aligned} S_a = f(s_{[x; y \, t]},c_{[x; y \, t]},o_{[x; y \, t]},r_{[x; y \, t]},p_{[x; y \, t]},a_{[x; y \, t]},n_{[x; y \, t]}) \end{aligned}$$

where $S _{a}$ is the soil attribute at a location; *s* is the soil type or other soil properties; *c* is the climate or climatic properties of the environment; *o* are the organisms, vegetation, fauna or human activity; *r* is topography or landscape attributes; *p* is parent material or lithology; *a* is the substrate age or the time factor, and *n* represents the spatial location. Variables *x*, *y* and *t* represent observations at a specific point in space and time, respectively. Soil attributes, such as carbon content, observed in limited sample locations can be used to predict soil carbon for the broader spatial area using their relationships with the ‘scorpan’ factors^22^.

A simple model of forest SOC, for example, could be that the total SOC is a function of carbon inputs from vegetation debris and outputs through decomposition. We can assume that vegetation litter-fall is proportional to vegetation density and its turnover. Quantitative information on these processes is rarely available at the spatial extent and scale required for estimates of national SOC stocks; alternatively, these processes can be captured by a set of proxies that are strongly related to these processes. The variation in forest carbon stocks is expected to be related to proxies of forest habitat conditions, in terms of climatic energy and water availability, and disturbance probability. We can also expect that relatively fine-scale variations in topography, combined with broad-scale orography and climate, will determine local temperature, radiation, and moisture conditions. Thus, we assumed that the combination of coarse-resolution gridded climate data and high-resolution topographic attributes provides critical information for predicting fine-scale variations in soil-forming processes and soil properties, including SOC.

The mean annual air temperature can be expected to be the most important predictor of variation in forest SOC observed at the forest inventory plot level. However, existing gridded datasets characterize the air temperature at a coarse spatial resolution, for example, 1 km^23,24^. In most mountain ranges, including the Central Himalayas, the mean annual air temperature is strongly correlated with elevation and can vary by hundreds of meters within a square kilometer. To capture some of the associated variations in air temperature, we used a high-resolution digital elevation model (DEM) as a predictor layer. Other terrain attributes derived at a fine spatial resolution (e.g., 30 m) can provide a detailed characterization of landforms and associated drainage and insolation patterns^25^. In addition, several other satellite-derived proxies of vegetation productivity, such as spectral vegetation indices, are available at fine spatial resolution. Previous attempts at spatially explicit modelling of soil information have typically used predictors related to terrain parameters derived from DEMs and remotely sensed surface reflectance^26^.

Various methods have been developed to generate spatial predictions of soil properties using spatially explicit covariates and georeferenced plot-level soil data. Over the last few decades, geostatistical approaches such as regression kriging^27^ and multiple linear regression^28^ have been commonly applied. However, recent research has shown that machine learning (ML) techniques, like random forest (RF), provide higher accuracy in predicting soil attributes than regression kriging^6,29,30^. Although ML models can be challenging to interpret^31^, they provide higher prediction accuracy than parametric models by fitting complex partitioning trees^32,33^. Thus, RF has become a popular approach for soil attribute modelling, including SOC stocks, and provides a higher accuracy in soil attribute modelling, including SOC stocks, and outperforms most other methods^26,34–36^.

A variant of RF, quantile regression forest (QRF), can estimate the full conditional distribution of a response variable, providing prediction intervals^37^. QRF can provide reliable estimates of uncertainty in the mapping of SOC and other soil properties compared to conventional regression kriging^38^. However, ML techniques, like RF, which handle high dimensionality and multicollinearity, are sensitive to spatial sampling design^39^. For example, modelling and predicting SOC over a large area is often based on plot data collected at regular intervals or in clusters, leading to spatial correlation. Failure to account for this spatial autocorrelation in the data can underestimate the prediction error^40,41^. Hence, it is important to consider the uncertainty introduced by the modeling approach, including spatial autocorrelation in the input data, when predicting soil attributes^22,42^.

A large range of potential sources of uncertainty exists in soil data analyses^43,44^. The uncertainty in the prediction of SOC is typically high in highly heterogeneous environments^45^. Quantifying the uncertainty in SOC estimates is a key requirement for evaluating the reliability of the results^46^ and for making the results usable. Broadly, uncertainties can be introduced due to: (1) inaccuracies in field observations, (2) poor model performance, and (3) inaccuracies in the input covariates. For robust quantification of prediction uncertainty, the magnitude of each source of uncertainty must be addressed. Furthermore, expressing prediction errors as maps helps in the robust evaluation of the spatial variability of uncertainty. However, it is often not feasible to quantify all sources of uncertainty due to the time and cost involved^47^. This is especially true when using SOC observations from field sampling and lab analyses from different projects that use different methods, standards, and sampling designs.

The primary aim of this paper is to provide the most accurate estimate of Nepal’s national forest SOC stock based on the available field observations and existing layers of covariates. To achieve this, we focused on building a statistical model for the spatially explicit prediction of current SOC using existing field observations of SOC and a range of variables that influence SOC, following the ‘scorpan’ model. In cases where the national scale SOC maps are unavailable, global soil maps can provide a useful overview of soil properties and conditions, although they may have some limitations in accurately representing national conditions. We also aimed to evaluate the global SOC products for the study area by comparing them with our predicted forest SOC map. With the additional considerations covering good practices in machine learning based spatial predictions, we demonstrated an improved approach for predicting forest SOC in highly heterogeneous countries.

### Materials and methods

#### Study area

Nepal is located in the Central Himalayas, with latitude extending from 26$^\circ$ 20$^\prime$ 53$^{\prime \prime }$ N to 30$^\circ$ 26$^\prime$ 51$^{\prime \prime }$ N, and longitude extending from 80$^\circ$ 03$^\prime$ 30$^{\prime \prime }$ E to 88$^\circ$ 12$^\prime$ 05$^{\prime \prime }$ E. The country covers a large elevational gradient from 59 masl in the southern Indo-Gangetic Plain to 8849 masl at the top of Mount Everest in the North. The pronounced topography and associated climate gradients/patterns sustain a highly diverse flora and fauna. With over 85% of Nepal characterized by mountainous landscapes^48^, this study area serves as an ideal test case to examine how well the distribution of forest SOC can be predicted in extremely heterogeneous environments.

#### Field data

**Figure 1:**
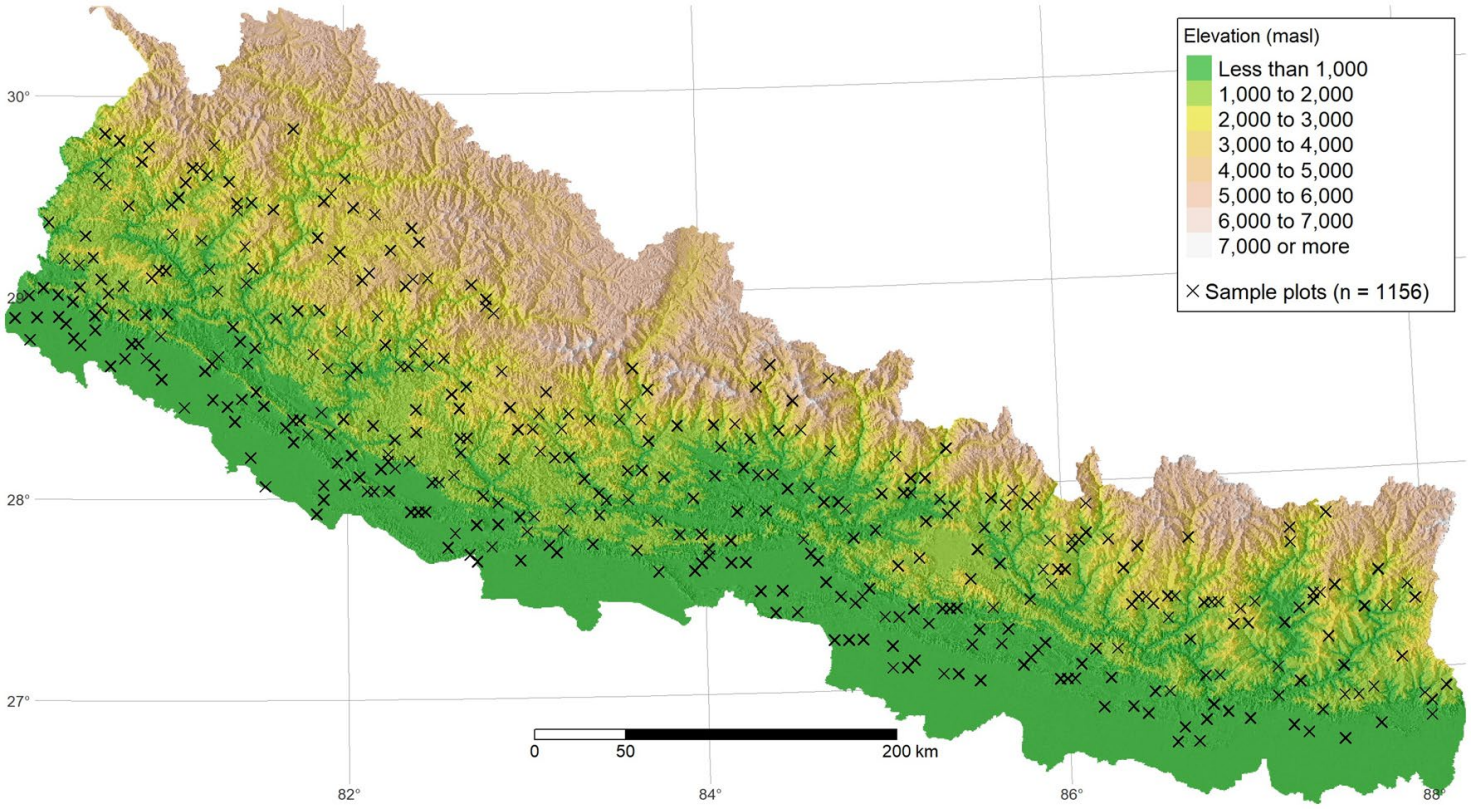
The distribution of field inventory plots with observations of SOC.

We used the existing field-based forest SOC stock data for topsoil (0–30 cm), which was collected as part of the national forest resource assessment conducted between 2010 and 2014. The details regarding the sample plot selection, soil sample collection, and lab analysis for SOC estimates are available in the field data set that has been submitted as a data paper, currently under review^49^. The plot-level estimates of forest SOC used in this study can be accessed at: https://doi.org/10.6084/m9.figshare.21959636^50^. The distribution of sample plots with SOC observations in the study area is shown in Fig. (1).

#### Selection of environmental predictors

We selected predictors based on the ‘scorpan’ framework (as listed in Table 1 and as maps in Supplementary Material Figure S1). Although predictors related to substrate age were not available, we expected that the other environmental variables such as topography, parent materials and soil types would provide constraints for the long-term potential or maximum amount of SOC expected at a location^51^. These environmental predictors are commonly used in spatial modelling of SOC^52^.

**Table 1:** Details of predictors selected for spatially explicit modelling of SOC.

Scorpan factors	Predictors	Description	Relationship to SOC
*s*	Dominant soil	21 types, soil and terrain database (SOTER) for Nepal^53^	SOC varies with soil type. E.g. clay soils have a much higher SOC than sandy loam^54^
*c*, *r*, *a*	Elevation	Digital elevation model, 30 m spatial resolution, ASTER^55^	Related to mean annual air temperature; higher elevation areas have a lower temperature
*o*	NDVI	Based on cloud-free Landsat 7 median composite image for ca. 2000, computed from cloud-free imagery acquired in the period 1999–2012^56^, version 1.6	NDVI is a proxy of landscape-scale photosynthetic activity forest^57^, and thus, long-term NDVI can be used as a proxy for net primary productivity^58^ and inputs of organic matter into the soil
*o*, *a*	Cost surface	Cost surface calculated using GRASS *r.walk*^59^. It represents the time in seconds needed to reach each grid cell from the road network based on national road data^60^. The slope (derived from DEM) was used as a friction surface	In a human-dominated landscape, the proximity of forest increases the likelihood of disturbance
*o*, *a*	Protected Status	Binary protected/non-protected area mask. Polygon layer from Dept of National Parks and Wildlife Conservation, Nepal, rasterised to 30 m pixel	The sites under the protection have lower disturbance and higher SOC stocks than comparable sites. For e.g. sites outside the protected area can lose twice the amount of SOC compared to protected sites in the humid tropical forest^61^
*o*	Distance from edge	Distance from the edge of the forest boundary towards the core using a forest mask^62^. This raster proximity map was derived by applying the *gdal:proximity* function^63^	The likelihood of disturbance is higher near the edge of the forest patch than near the core
*r*	Slope	Slope gradient derived from the digital elevation model	The erosion potential increases with slope gradient, and thus steep slopes are likely to have shallow soils. The shallow soils have a small water storage capacity and generate runoff more frequently than deeper soils
*r*, *c*	Wind exposure	The average ‘Wind Effect Index’ for all directions using an angular step. A dimensionless index. Values below 1 indicate wind shadowed areas, whereas values above 1 indicate wind-exposed areas. Derived using the SAGA-GIS package^64^	Considers aspect. Sites North-facing slopes can have three times higher SOC levels than South-facing slopes^65^
*r*, *c*	TWI	Topographic wetness index (TWI) indicates the potential for water to accumulate. A high index value indicates the high water accumulation potential. Unitless. Computed using the SAGA-GIS package^64^	Higher soil moisture availability favours plant productivity and thus more carbon inputs to soil
*r*	TPI	Topographic position index (TPI) compares the elevation of each cell in a DEM to the mean elevation of a specified neighbourhood around that cell. A 100 m × 100 m neighbourhood was used. Positive TPI values represent locations that are higher than the average of their neighbourhood window (e.g. ridges), negative values are lower (e.g. valleys), and flat areas are close to^66^	Curvature controls the water redistribution and substrate thickness and is an important determinant of SOC^65^
*p*, *a*	Parent materials	Soil and terrain database (SOTER) for Nepal^53^ provides a parent material type map representing eleven types based on lithology	Parent materials determine the soil properties and determine SOC distribution^67^

The spatial resolution of all predictors is 30 m.

#### Prediction of forest SOC stock and uncertainty

Quantile regression forest (QRF) was used to model spatial variation in forest SOC as a function of gridded predictors at 30 m spatial resolution. We fitted the QRF model using functions implemented in the R package quantregForest^68^ and evaluated the importance of the gridded variables. This model follows a geostatistical approach that integrates field measurements, predictors related to terrain, climate and computer algorithms to develop models to predict values of interest for the study area^69^. All analyses were performed using the R software package^70^.

After fitting the QRF, prediction uncertainties were quantified. Uncertainty in digital soil maps comprises four major components related to uncertainties in the model, spatial distribution of sample plots in geographic space, soil data and covariates used in the model. Here, we focused on two major sources of uncertainty: (a) model uncertainty (variability in prediction) and (b) sensitivity (variability due to sample plot distribution). Additional uncertainties are related to input soil data and covariates, which we have not addressed in this paper due to the unavailability of information to quantify their contribution to the total uncertainty.

Spatial cross-validation was used to evaluate the effect of sample plot distribution on the accuracy of predicted forest SOC. The clustered sampling design field observation of forest SOC used in this study^62^ is common in national forest inventories to collect tree and soil samples^71,72^. In such cases, random sampling for data partitioning into training and test data is not ideal because it can introduce over-fitting due to spatial autocorrelation. In these cases, spatial k-fold cross-validation is recommended^73^. Evaluation of different strategies for evaluating map accuracy with clustered data show that different strategies are necessary to address varying levels of clustering, but blocked spatial cross-validation works best for strong clustering^74^. The k-fold cross-validation (CV) procedure, where k is the number of groups the data is split into, was used to evaluate model performance. Partitioning data within spatial CV considers the cluster or block information and iteratively leaves out all plots belonging to a randomly selected group of clusters. Here, the *CreateSpacetimeFolds* function in the CAST package^75^ was used to create spatial folds for CV by specifying a plot cluster id as the spatial units of sample plots. This data partitioning provided two lists for model training and model validation used as *index* and *indexOut* in caret’s *trainControl* function^76^. Each resampling iteration for model training and validation used the list of elements representing the training and validation sets. The details on the quantification of the two major prediction uncertainties are as follows:

*(a) Model uncertainty* QRF provides the full conditional distribution for each of the prediction locations, allowing the examination of variability in the predicted forest SOC across the entire covariate space. We estimated model uncertainty using the standard deviation (SD) of this conditional distribution. A QRF model was fit with a 10-fold spatial CV and selected a model with the smallest Root Mean Squared Error (RMSE). Then, the conditional distribution was predicted using fitted QRF. In this step, the gridded mean and SD of SOC were predicted by taking into account the effect of sample plot design (sensitivity) since the final QRF was selected based on spatial CV. RMSE was used to examine the average prediction error of the model when predicting the independent observations set aside for testing:

3 $$\begin{aligned} RMSE = \sqrt{\frac{\sum _{i=1}^n{(y_i - \hat{y}_i)^2}}{n}} \end{aligned}$$

where $y_i$ is the observed value and $\hat{y}_i$ is the predicted value for the ${\textrm{ith}}$ observation.

*(b) Sensitivity* The sensitivity refers to the uncertainty in model prediction due to the spatial sampling design. Using the covariates listed in Table 1, we fitted a QRF model with a 10-fold spatial CV and ten realisations of the predicted SOC maps. This method of CV is also termed as 10-fold Leave-Location-Out cross-validation^75^. Then, we derived the sensitivity of the model due to the spatial sampling design as the SD of 10 realisations of predicted SOC.

We then combined both sources of error, model uncertainty and sensitivity, and expressed as a percentage:

4 $$\begin{aligned} Percent \ Error = \frac{(Model \ Uncertainty + Sensitivity)}{Mean \ Prediction} \end{aligned}$$

where the predicted mean and uncertainty of the model are the predicted mean and SD of conditional distribution from the fitted QRF, respectively. Sensitivity is the standard deviation of SOC predicted using a 10-fold spatial CV.

We computed the total national forest SOC and standard error (SE) by summing all predicted SOC and SE for all individual grid cells. The standard errors for all grid cells were aggregated and expressed as a relative percentage error.

To compare the performance of random CV with that of spatial CV, we fitted a QRF model using a 10-fold random CV with the *createFolds* function in the R package caret^76^. The presence of spatial autocorrelation in input data leads to the autocorrelation of residuals, which can influence the modelling results and interpretation of the results^77^. To assess this, we computed the semivariance, a measure of average dissimilarity between field observations collected at locations separated by a certain distance, for the residuals obtained from both spatial and random CV approaches. An experimental semivariogram was used to examine the variance between pairs of data over a varying range of distances. The R package geoR^78^ was used to examine the semivariogram of the residuals from both CV approaches. Semivariance was computed up to a maximum distance of 25 km, and the default 99 random permutations were used to allocate data to the spatial locations, compute the empirical variogram for each permutation, and derive the envelope for the empirical variogram.

Representativeness of the prediction space covered by training data We evaluated the representation of the prediction space in the study area by using input field training data and the spatial prediction model used for estimating model uncertainty. This analysis is important because when predicting forest SOC over a large geographical region, some areas or part of the multivariate predictor space may be under-represented by training data. In such cases, the model fitting does not consider the characteristics of these under-represented areas, and the prediction for those areas can be highly uncertain. The AOA (Area of Applicability) concept can be used to evaluate this source of uncertainty^79^. The approach involves (a) calculation of the Dissimilarity Index (DI), which is the minimum distance to the training data in the multivariate predictor space and then (b) using the $0.95_{\textrm{th}}$ quantile cut-off of the DI in the training data (on the multiple DI generated for each site with cross-validation). AOA and DI were calculated using the *aoa* function in the R package CAST^75^. The AOA analysis produces a binary map with ‘1’ representing the area inside the AOA, where we can have confidence in the model prediction, while areas with ‘0’ represent the area outside the AOA, where the model prediction is uncertain.

#### Comparison of forest SOC predictions with existing global SOC estimates

We compared forest SOC predicted by this study against two existing global data sets. The first global dataset provides estimated SOC stocks in topsoil (0–30 cm depth) at 1 km spatial resolution by FAO^80^. This data was derived by compiling and harmonising existing national scale SOC estimates. For the countries that do not have national mapping of SOC, spatial modelling was done using publicly available data. In the case of Nepal, SOC estimates for 6000 sample plots collected between 1990 and 2000 for croplands, not forests, were used for spatially explicit mapping. The second global dataset, SoilGrids250m 2.0^26^, also provides estimates of SOC for topsoil (0–30 cm depth) but at 250 m spatial resolution. It uses available observations from the World Soil Information Service (WoSIS) Soil Profile Database and a range of environmental covariates for spatial prediction of SOC globally. The spatial map of both of these global data sets covering Nepal is presented in Supplementary Material Figure S3. The spatial resolutions of these existing global datasets are much larger than the SOC predicted by this study (30 m). To deal with the differences in the spatial resolution between my model prediction and these global datasets, we aggregated predicted forest SOC to the larger grids ($\sim 1$ km and $\sim 250$ m) using the mean function and then compared the values for all the resampled predictions against the subset of existing global datasets covering the study area.

### Results

#### Spatial distribution of forest SOC stocks

**Figure 2:**
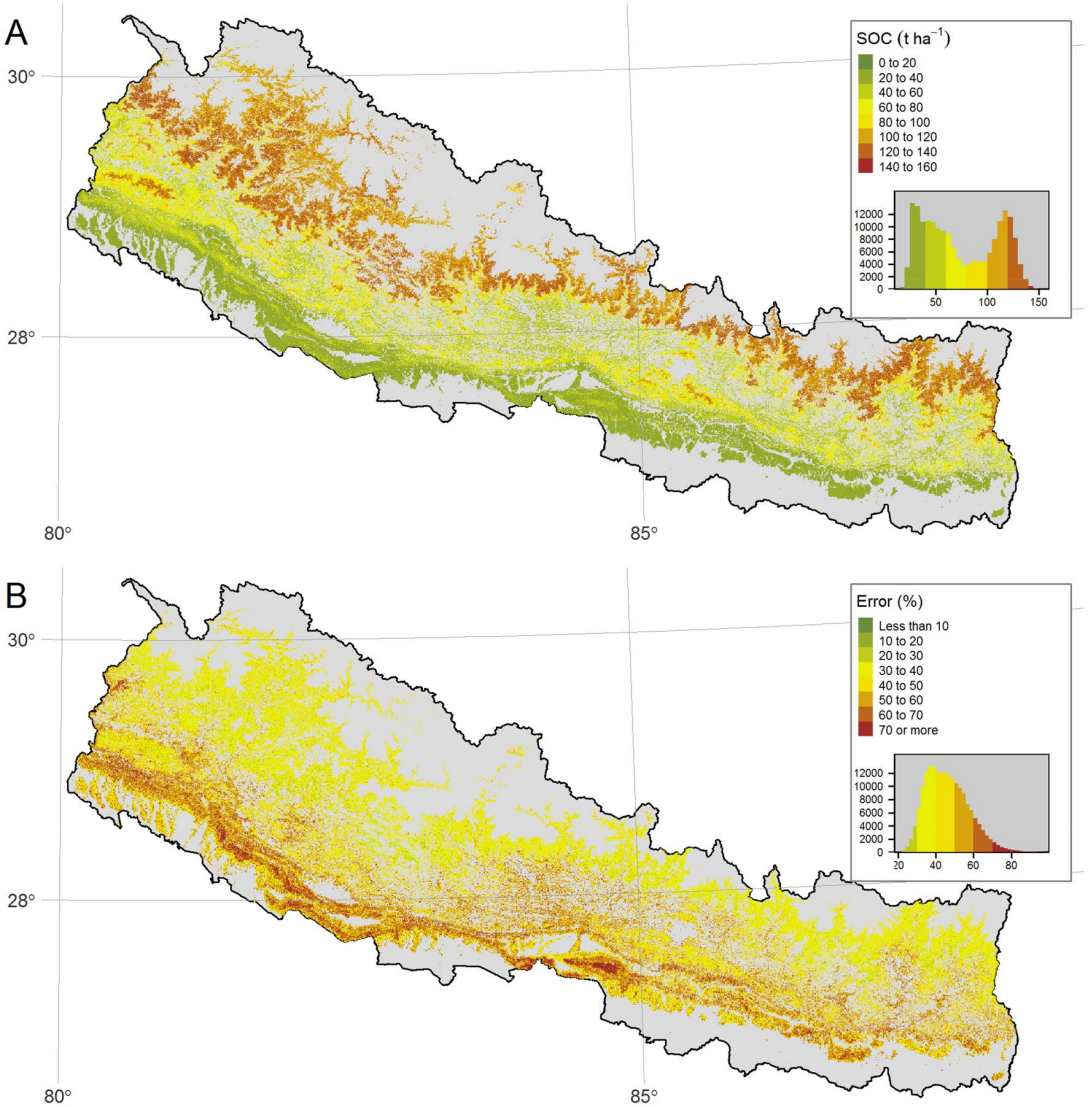
Predicted SOC stocks and the percentage prediction error based on the 10-fold CV. Panel (**A**) shows the mean SOC predicted using quantile regression forest (QRF), and Panel (**B**) shows the prediction error expressed as a percentage. The histogram in the legend shows the distribution of the data presented on the map. All figures were created using tmap package^81^ in R^82^.

The spatial distribution of predicted SOC and prediction error showed some association with major physiographic regions (Fig. 2). The distribution of predicted forest SOC showed an increase along the elevational gradient, which increases from South to North across Nepal. This variation follows the distinct physiographic regions occurring at varying elevational ranges. The predicted forest SOC showed a bimodal distribution, indicating the presence of two different groups represented by the two peaks of data, with relatively low SOC values (approx. 10 t C $\hbox {ha}^{-1}$) in lowland forests and relatively high SOC values (approx. 125 t C $\hbox {ha}^{-1}$) in high elevation forests. Along the elevational gradient, increasing from South to North, the results showed a drop in forest SOC in the middle mountain region of Nepal (Fig. 2A Histogram). The results also showed that the forest SOC reaches maximum in the 3000–4000 masl elevation range of > 4000 masl. The locations with the highest prediction error of forest SOC are predominantly located in the lower foothills (Fig. 2B). The total estimated SOC in topsoil (0–30 cm) and the standard error as a percentage of the total estimate for the entire forested area of Nepal was estimated at 494 Mt C (SE = 3.46%), an average of 75 t C $\hbox {ha}^{-1}$.

#### Evaluation of uncertainty

**Figure 3:**
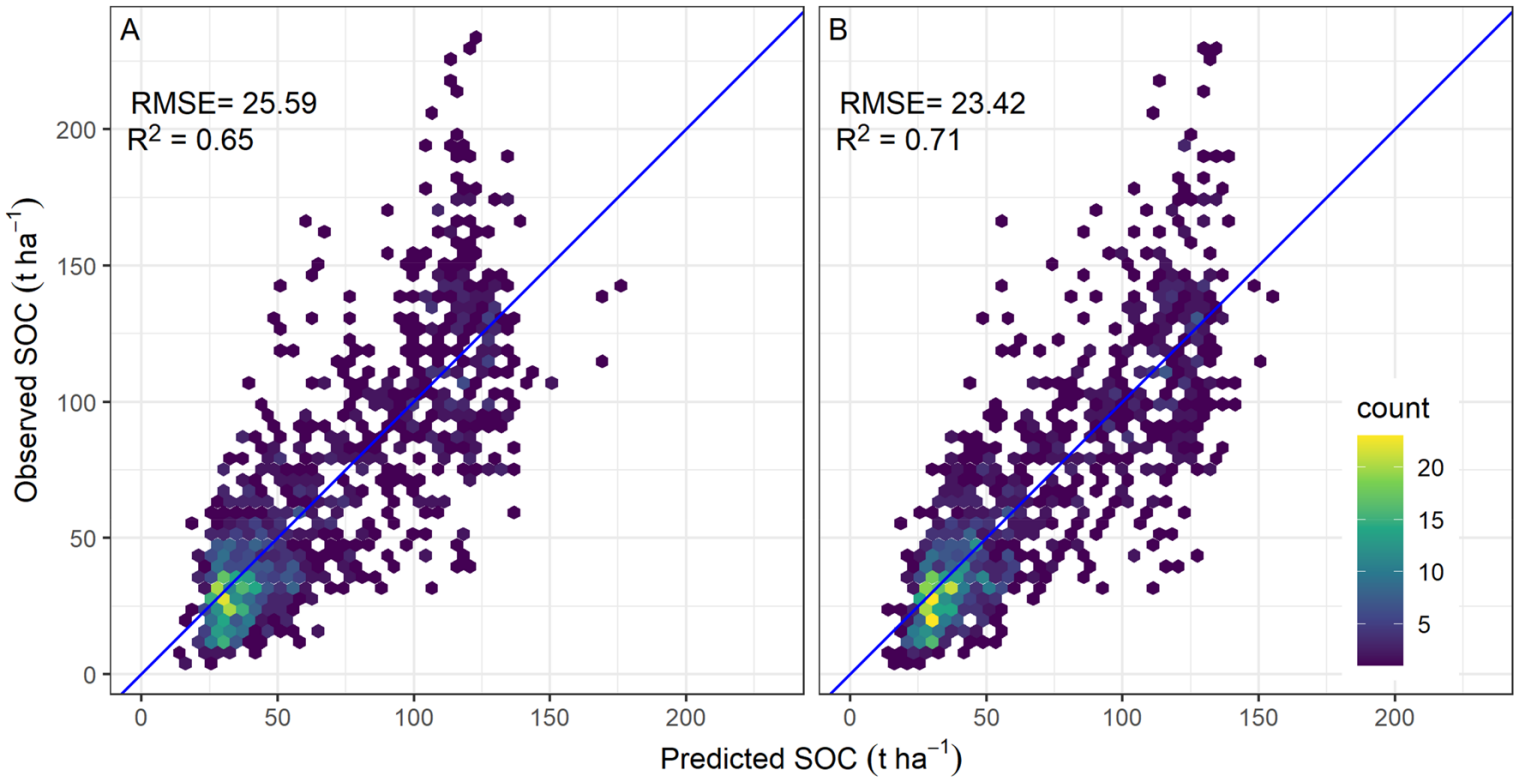
Comparison between observed and predicted SOC using K-fold CV for all input plots (1156). Panel (**A**) compares observed SOC against the prediction based on 10-fold spatial CV and Panel (**B**) against the prediction based on 10-fold random CV. RMSE in t $\hbox {ha}^{-1}$ and $\hbox {R}^{2}$ represents the coefficient of determination. Figure was created using ggplot2 package^83^ in R^82^.

Despite reasonable prediction accuracy, the spatial CV showed lower agreement with observed SOC compared to random CV indicating pessimistic map accuracy results (Fig. 3). Models using both CV approaches underestimated SOC for plots with more than 150 t $\hbox {ha}^{-1}$. However, only 4% of the sample plot observations were in this high SOC range. The model error component, as quantified by the standard deviation of 10-fold CV prediction using QRF (Supplementary Material Figure S2A), was much larger than the error due to the sampling design (sensitivity) (Supplementary Material Figure S2B). The model uncertainty was larger than the uncertainty due to the sampling design (sensitivity). The higher model uncertainty can be interpreted as the effect of weak relationships between SOC and environmental predictors that are built into the QRF model, which indicates that the input data only partly capture the processes that control spatial variation in SOC over the highly heterogeneous study area. The low elevation forests have lower forest SOC than high elevation forests. Therefore, the relative error expressed as a percentage is much larger in low-elevation forests than in high-elevation forests. Due to the differences in the range of forest SOC with high stocks in high elevation forests, the larger absolute prediction errors occur for plots with high SOC, mostly at high elevation.

**Figure 4:**
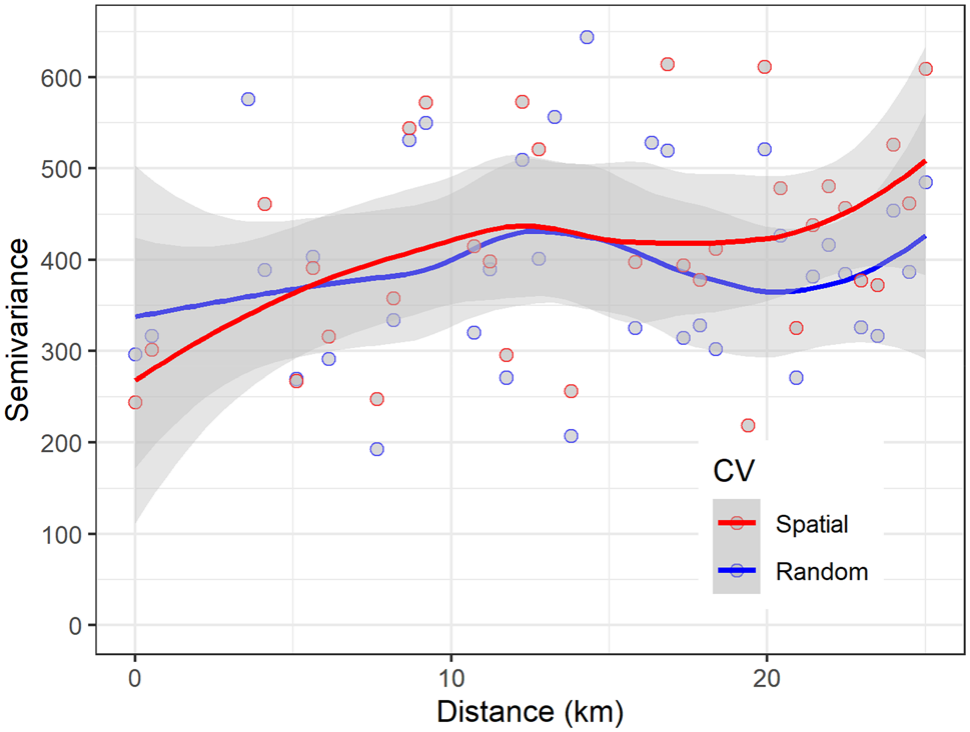
Variogram of residuals from QRF models using spatial CV and random CV. The points in the plot show semivariance at 50 regular distance bins, and envelopes show the variability of the empirical variogram. Figure was created using ggplot2 package^83^ in R^82^.

The semivariance of model residuals showed a similar pattern for both random and spatial CV (Fig. 4). The shaded area represents envelopes for the empirical variogram derived by random permutation of the residuals. Thus, the envelope represents the situation of no spatial autocorrelation. The observations in the variogram show the semivariance, which is the squared differences between residuals as a function of the distance between sample sites^84^. Following the established rule in geography that objects in closer proximity are similar, we expect the semivariance to increase with distance and stabilise at a certain distance. Ideally, we are interested in examining the sill (the semivariance at which the variogram first flattens out) and the range (the distance at which the variogram first flattens out). We typically expect observations at distances within the range to be correlated, but plot-level forest SOC observations used in this study did not show a strong correlation. Along the entire distance lags, the semivariance of residuals for the model fitted using both spatial and random CV have largely overlapping confidence intervals. The two semivariograms are practically flat, showing that the residuals contain very little spatial autocorrelation in both cases, spatial and random CV. The largely ‘flat’ semivariogram of the residuals shows that the model has captured most of the spatial variation.

#### Area of applicability of spatial prediction model

**Figure 5:**
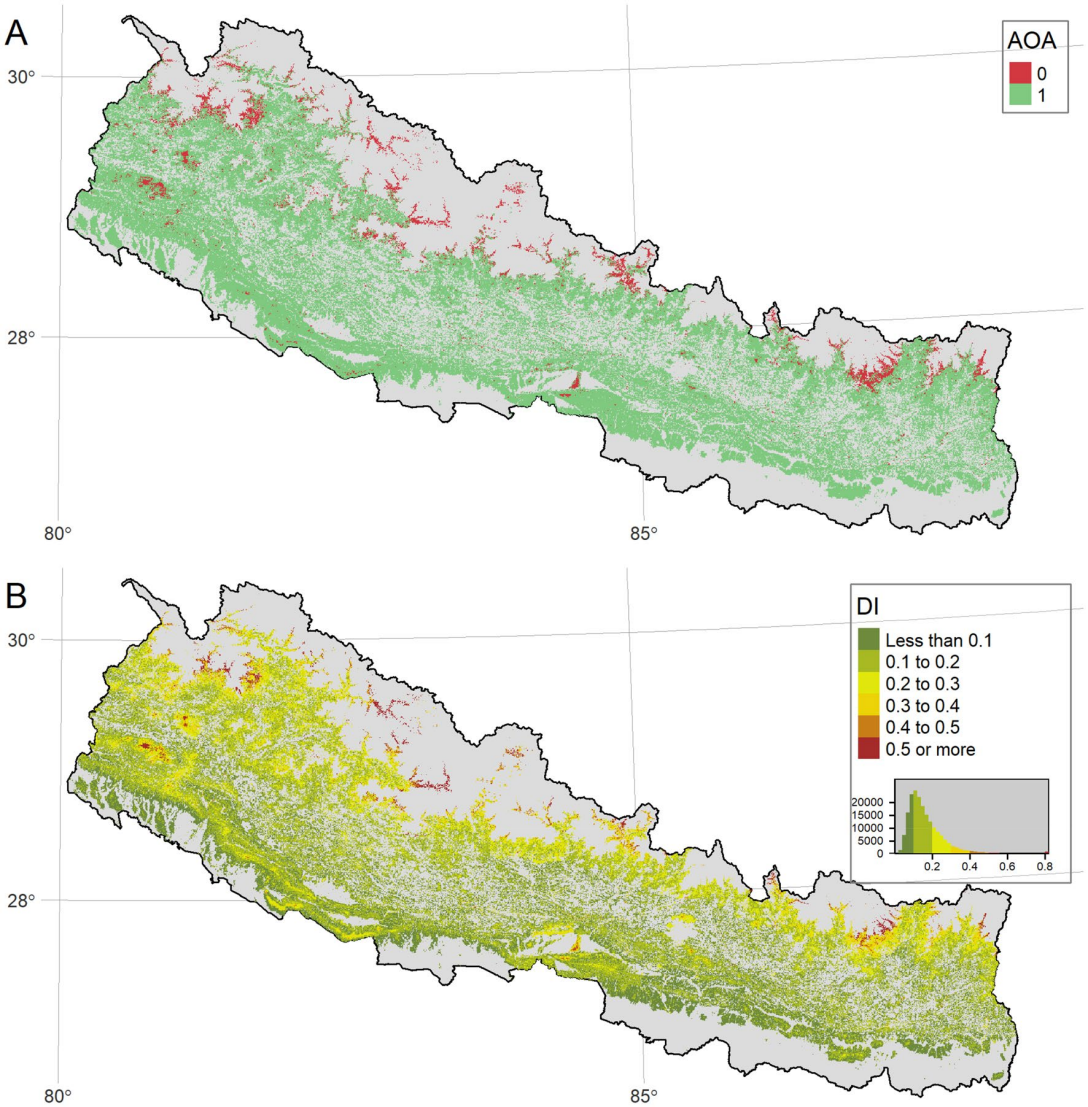
Area of applicability plot. Panel (**A**) shows the Area of Applicability (AOA), and panel (**B**) shows the Dissimilarity Index (DI) for the forest SOC prediction model. All figures were created using tmap package^81^ in R^82^.

The spatial distribution of both Area of Applicability (AOA) and Dissimilarity Index (DI) showed that the prediction space not represented by training data is a small fraction of the total forest area and concentrated in the highest elevation zones (Fig. 5). AOA with value 0 indicates the sites that are not represented by training sample plots. The obvious association between DI and AOA exists as the binary AOA is derived from the DI values. The spatial pattern of DI values also shows that the DI with high values are particularly concentrated at high elevation forests. Further, high DI values are also observed in the Churia hills, located in the low elevation zones of Southern Nepal. The forest stands with large values of DI correspond to AOA = 0 values and indicate that these stands are not represented by the training data used in modelling forest SOC.

#### Variable importance

**Figure 6:**
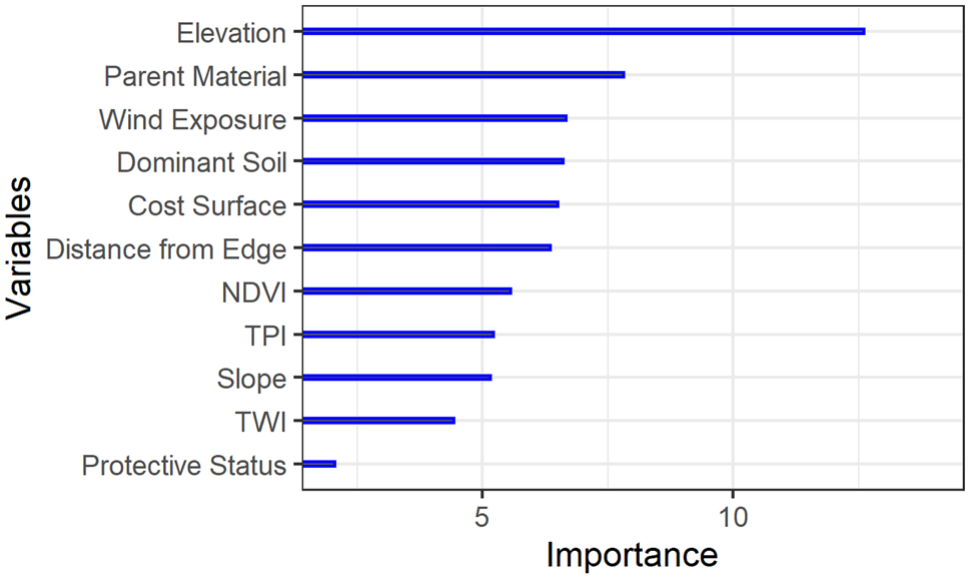
Variable importance of the SOC prediction model. Figure was created using ggplot2 package^83^ in R^82^.

The input covariates in the QRF model used for forest SOC prediction showed varying relative influence on the predicted SOC (Fig. 6). The importance values for each variable represent the Mean Decrease Accuracy and express the decrease in model accuracy when each variable is removed. The higher values of importance indicate the higher importance of the variable to the model. The elevation showed the highest relative importance, the protected status showed the least importance, and the relative importance of the remaining variables varied marginally in the QRF model. Though elevation, a proxy of climatic conditions, was observed as the most important variable, parent material, dominant soil, and terrain attributes are similar and of intermediate importance. The partial dependence plots also confirmed the the marginal effect of each variable on the prediction of forest SOC (Supplementary Material Figure S7) The order of the variable importance in the model thus is relatively consistent with the inputs of the ‘scorpan’ framework.

#### Comparison of model predictions with existing global SOC data products

**Figure 7:**
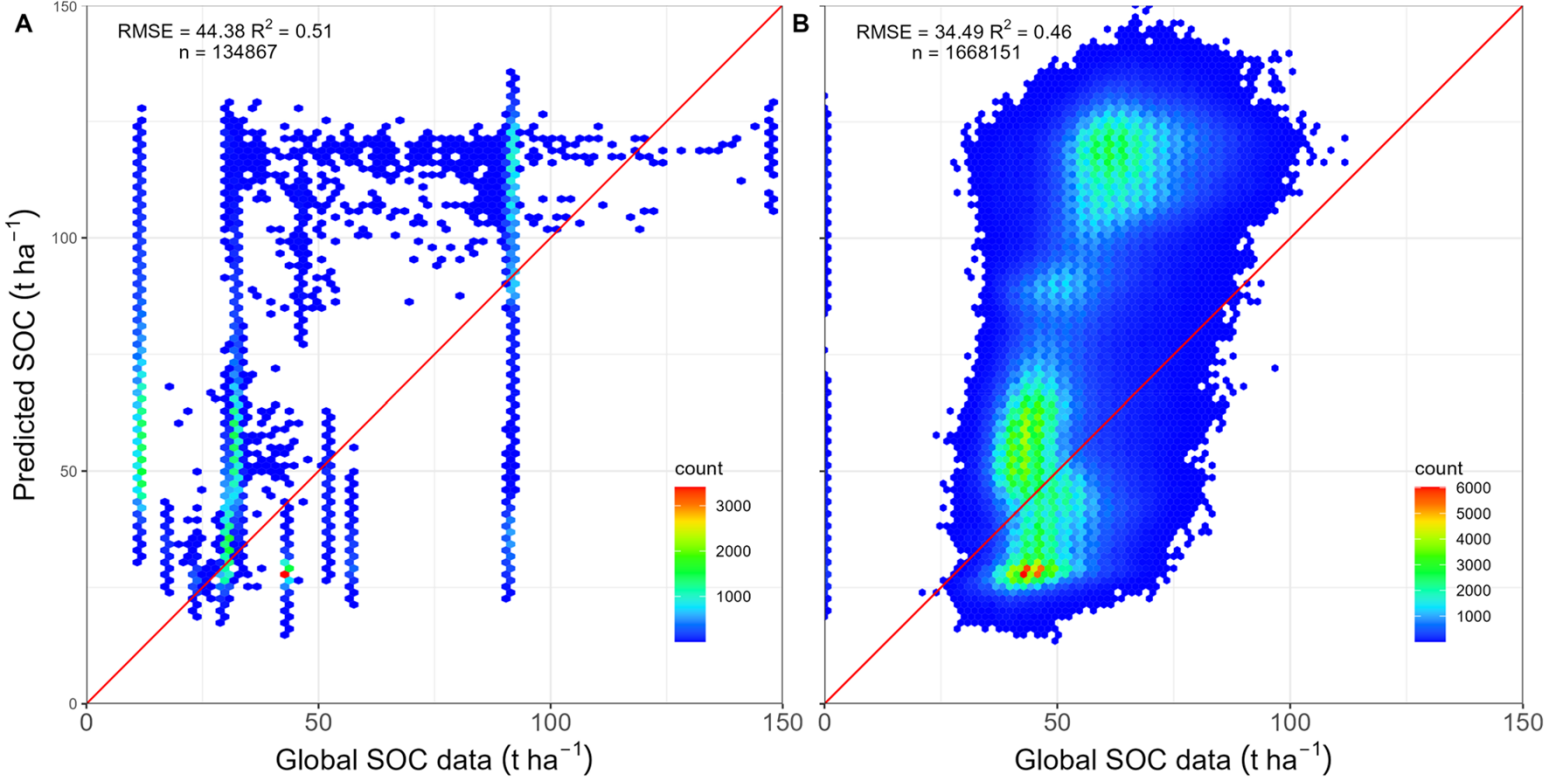
Comparison of predicted forest SOC against existing global-scale data products. The colour gradient represents the elevation of the input plot location. Panel (**A**) compares predicted SOC against existing predictions by GSOCmap^80^ and (**B**) against SoilGrids250m^26^. The Red line is the 1:1 line, and RMSE is in t $\hbox {ha}^{-1}$. Figures were created using ggplot2 package^83^ in R^82^.

Large deviations were observed when the predicted forest SOC was compared with two existing global SOC data products (Fig. 7). Particularly, the deviations differed significantly across the elevational zones, with the highest elevation zones having the largest deviation between global and national observations (Supplementary Material Figure S4). Field observations and predicted forest SOC stocks were compared with existing global SOC data products, revealing significant deviations, especially in high-elevation forests (Supplementary Material Figures S5, S6, respectively).

### Discussion

The primary aim of this paper is to provide the most accurate estimate of Nepal’s national forest SOC stock. The total SOC in topsoil (0–30 cm) for the forested area in Nepal was estimated at 494 Mt C (SE = 16). Using the same set of field data, Nepal’s national forest resource assessment derived the total forest SOC as 422 Mt C by multiplying the average forest SOC density with the total forest cover area^62^. The aggregation of SOC estimates in existing global data over forested grids of Nepal shows much lower estimates, i.e. 58 Mt C^85^ and 6 Mt C^80^. This significant variation is driven by the underestimation of forest SOC across Nepal (Fig. 7). The spatial distribution of predicted SOC stocks showed that the largest forest SOC stocks occurred in forests of the high elevation zones (Fig. 2A). This spatial distribution pattern is consistent with decomposition rates tending to increase with mean annual air temperature, and thus long-term accumulation of organic matter (e.g. leaf litter, wood debris, twigs) to be higher in the cold climatic regions of the mountains than in the tropical lowlands. This finding agrees with other studies showing the negative relationship between air temperature changes and sensitivity of organic matter decomposition^86^. In the context of climate change, the understanding spatial distribution of SOC in the mountain is important because it is very vulnerable to warming. Increasing air temperature leads to an exponential increase in organic matter decomposition^12,87^. In the case of mountain soils, the projected elevation-dependent warming^88^, combined with the presence of a high portion of the carbon in a labile fraction^12^, are projected to experience a large loss in SOC in high latitude areas^89^. Previous studies have observed large SOC stocks in forests compared to other land-use in the mountainous region^52^.

The average forest SOC for 0–30 cm depth, estimated from the national forest inventory plot (67.14 t C $\hbox {ha}^{-1}$)^62^ is comparable to the average forest SOC for Bhutan (64 t C $\hbox {ha}^{-1}$)^90^ and Swiss forests (76 t C $\hbox {ha}^{-1}$)^91^ for 0–30 cm depth. SOC in mountain forest areas is likely to play a significant role in the global carbon balance context^92,93^ and deserves attention in the light of projected climate change impacts. The spatially explicit prediction of SOC and the uncertainty presented in this study provide a benchmark map that provides a basis for assessing the expected impact of future climate changes in the Central Himalayas.

The Central Himalayas are among the most rugged regions on Earth, and the elevational gradient in the study area is one of the largest globally. The results showed elevation as the most important predictor of the spatial variation in forest SOC (Fig. 6). This trend is due to the gradient in mean annual air temperature, which typically decreases by $0.5\,^{\circ }\hbox {C}$ for every 100 m increase in elevation in Nepal^94^. The observed increase in forest SOC stocks with elevation is consistent with elevation being a proxy for mean annual air temperature and, hence, lower decomposition rates at higher elevations^95^. Using elevation as a predictor is recommended for spatial modelling of SOC^96^ because, besides the elevational gradient, the digital elevation model captures variation in the topographic position, which also controls the spatial distribution of SOC^9,97^. As SOC is a function of litter production (inputs) and decomposition rates (outputs), the high elevation forests combine relatively high productivity with relatively low decomposition rates and thus high SOC stocks. However, above the alpine treeline, air temperatures decline along with plant growth, litter production and decomposition, resulting in expected lower SOC. Nevertheless, this trend cannot be confirmed in this study as the forest inventory data do not represent conditions above the treeline.

The results showed that wind exposure is an important predictor of forest SOC after elevation and parent material (Fig. 6). At the alpine treelines, the effect of wind on forest SOC varies with site conditions. For example, depending on the slope and curvature, the windward sites experience higher wind erosion potential^98^ and low soil moisture^99^, while leeward sites often retain snowpacks for a longer duration which favours the survival of tree seedlings^100^. The wind exposure variable is expected to have captured some of this topography induced variability in solar radiation, precipitation distribution, soil erosion, and hence, forest SOC. Some covariates, such as TWI, which show the potential water redistribution in the landscape based on topographic features, had low importance for the SOC prediction model. In this highly heterogeneous study area, topographic positions with higher potential wetness may not have an abundant water supply. For example, deep gorges in high mountains may have high TWI values but scarce rainfall.

Proxies of human disturbance probability (cost surface and distance from forest edge) showed relatively high variable importance (Fig. 6). The findings agree with a general observation that disturbances such as fire and tree cutting, tend to negatively influence SOC stocks^101^. Human disturbances, such as fuel-wood and leaf-litter collection, livestock grazing and burning reduce the deposition of leaf-litter and coarse woody debris and hence SOC accumulation. The input variables captured the expected higher intensity of human disturbance to forests in proximity of the forest edge or road network. The observed increase in forest SOC with elevation can be partially attributed to the long-term carbon accumulation but also to lower human-induced disturbances^102^. The elevation gradient also served as a proxy of human-induced disturbance intensity, as with increasing elevation, there is a decrease in population density and accessibility. In the middle-mountains of Nepal (< 3300 masl), road construction activities are the predominant trigger for landslides^103,104^ which would also affect forest SOC. The strong correlation between elevation and mean annual temperature possibly confounds the variation in human-induced disturbances along the temperature gradients. However, the low elevation region of the study area with higher average temperature and population density would represent a contrasting situation compared to the rest of the country. The predictor of binary protected status was found to have low importance indicating that the variability of SOC distribution was not necessarily dependent on whether a site is within or outside the protected area. This is likely the effect of high environmental heterogeneity of the study area combined with highly varying protected status, resource use patterns and disturbance history. Given the lack of consistent observations on disturbances, the study showed that the use of proxies of disturbance likelihood could capture some proportion of disturbance impact on forest SOC.

The spatial representation of the prediction error showed that the pattern of errors varied by region. Despite having a large number of sample plots, the lowermost foothills had the highest percentage prediction error (Fig. 2B). The lower foothills, called Churia hills in Nepal, have predominantly dry, rocky landforms and dissected landscapes with relatively high erosion rates^105^ and low forest SOC. The larger uncertainty expressed as percentage error in Churia is thus the combined effect of lower average SOC stocks (Fig. 2A) and higher relative model uncertainty (Supplementary Material Figure S2). Even for the similar absolute error for forest SOC across elevation zones, the percentage error will be higher in the low elevation forests with low SOC. Other metrics for quantifying errors, such as mean absolute errors that require individual observed and predicted values, are not relevant here as the standard errors of prediction were derived and later expressed as the percentage of the average SOC for each grid-cell. High elevation forest sites showed higher model uncertainty which was derived as the standard deviation (SD) of predicted conditional distribution (Supplementary Material Figure S2A). The SD widened for the larger predicted values of forest SOC at the higher elevation. The mountains have large topoclimatic variability introducing high spatial variability in environmental conditions and thus in forest site productivity^106^ and forest SOC stocks^107^. We can expect higher variability in SOC estimates over smaller distances for this highly heterogeneous region. Large spatial variability of soil properties is a significant source of uncertainty for soil carbon prediction in mountainous regions^107^. Terrain complexity in the mountains can also introduce uncertainty in the input predictors, particularly for satellite-derived variables^108^, such as NDVI due to terrain effects such as illumination^109^ and viewing angles^110^.

The differences in uncertainty between spatial and random cross-validation were marginal indicating an insignificant effect of clustered sampling design on the prediction of forest SOC (Fig. 3). Earlier studies modelling diverse ecological attributes such as air temperature, soil water content^73^, land cover, leaf area index^111^, and terrestrial radiation^112^ have shown that the random CV strategy considerably underestimates the model error. For example, a large reduction in the agreement between predicted and observed estimates of aboveground biomass (AGB) in the Congo basin was observed when comparing spatial CV against random CV^40^. As the authors used the clumped patches of raster grid cells with AGB estimates as the model input, input training data consisted of contiguous grid cells with higher homogeneity among nearby cells, and hence high autocorrelation was expected. For the present study, since plot-based SOC estimates showed high spatial variability in the mountainous region over shorter geographical distances, the cluster design did not significantly affect geographical proximity. Further, in clustered sample design adopted for the forest inventory sampling protocol, the distance between plot clusters is large enough (i.e. $\ge$ 4 km) such that we cannot see the significant autocorrelation. The variogram analysis also confirmed the lack of a strong autocorrelation in model residuals (Fig. 4). The similarity in the comparison between the semivariance of residuals derived from random and spatial CV confirmed the highly variable nature of SOC over a shorter distance. Thus, for the highly heterogeneous sites, forest SOC estimated using clustered plots separated at a smaller distance (e.g. 200 m) does not show strong autocorrelation.

Area of Applicability (AOA) analysis revealed that the input training data for the model fitting largely represents the environmental variability across most of the study area. However, high elevation forests, which constituted the areas with the largest Dissimilarity Index (DI) (> 0.3), represent relatively small areas that fall outside the AOA. The predominance of these areas outside AOA in locations near and above the alpine treeline highlights the impact of the limited number of training sample plots in that elevation range of the study area. Poor accessibility, a result of the rugged mountain terrain, is one of the key challenges in field sampling, resulting in a smaller number of sample plots at high elevations. The clustered sampling design used in this study, with 150 m between plots and $\ge$ 4 km between clusters, may not be suited to capture fine-scale heterogeneity of topoclimatic conditions. Another potential issue that contributes to these locations outside AOA can be related to the inclusion of bare or non-vegetated locations in the forest mask due to potential misclassification of forests at the high elevation sites. Although the area outside AOA is much smaller than the country’s total area, one option is to include SOC sampling along with the finer elevational transect that is representative. However, sampling in the high elevation areas poses considerable logistical challenges due to extreme weather conditions and demanding terrain. Many of these areas will not be accessible for field sampling, even with large resources and efforts.

The cross-validation of the SOC prediction showed a reasonable accuracy of the model. A comparison of field observations of SOC in forest inventory plots against two existing global-scale products (Fig. 7) revealed that the prediction model using available forest SOC data has enabled us to make an informed quantification of SOC across Nepal (RMSE of 25.59 compared to 45.49 and 39.61, Figs. 3, 7 respectively). The magnitude of deviation between predicted forest SOC from this study and existing global datasets varied with elevation zone. In particular, the high elevation region showed the lowest agreement between predicted SOC from this study and existing global SOC data products. The varying regional bias between field plot measurement and global data has reported for forest biomass^113^. Despite a reasonable accuracy of spatially explicit SOC maps produced by some initiatives on a global scale^26,114^, a comparison of global SOC predictions against the predicted SOC as well as field observed SOC showed large bias, as expected. Our result agrees with reported underestimation of up to 40% in global data compared to local field-based estimates from the USA and Europe^115^. This observation led to the suggestion that the global data sets could not account for the fine scale spatial variation in bulk density and organic carbon concentration^115^. A similar observation was recorded in the case of Tibet, whereby the global datasets both over and underestimated SOC, depending on the sub-region, with a larger bias predominantly found in sites of complex topography^52^. Similarly, we found that the global products underestimated forest SOC in low elevation zones while overestimated in high elevation zones. For example, the RMSE between predicted SOC and global datasets was almost three times larger (15 t $\hbox {ha}^{-1}$ and 55 t $\hbox {ha}^{-1}$) in the high elevation zones (> 3000 masl) compared to lower elevation zones (< 1000 masl) (Supplementary Material Figure S5). The large bias limits the direct use of global SOC maps to make reliable inferences on the variability of SOC, particularly for a smaller geographic region and countries with high environmental heterogeneity. The bias in the global prediction is expected as global-scale predictions often rely on limited training datasets available for training prediction models^80^. Field-based SOC sampling is often focussed on arable land for agricultural applications, which results in relatively few observations of forest SOC. The observed large deviation in existing global SOC estimates from the field-based observations suggested caution is required when using global datasets to make inferences about the current stocks and changes in SOC over the mountainous region. This disagreement also implies the need for the global models to reduce the uncertainty in the high elevation forest SOC estimates by expanding observations from the data gap regions as a priority.

### Conclusions

In this study, we aimed to quantify the spatial distribution of forest soil organic carbon (SOC) stocks in the Central Himalayas by predicting their spatial distribution across Nepal. Our predictive model, informed by a range of spatial covariates, resulted in spatially explicit predictions of forest SOC with associated prediction uncertainty. Our study provides benchmark SOC estimates for the Central Himalayas, one of the largest elevational gradients on Earth, and highlights regional variation in deviations between global and national estimates. By combining our spatially explicit SOC estimates with those of another carbon pool, such as forest aboveground biomass, we can achieve a more comprehensive accounting of forest carbon. This study offers important implications for REDD+ reporting, forest management, and ecological applications. Our approach, which incorporated good-practice in machine learning including quantification of uncertainty introduced by model error and spatial sampling design, offers a promising method for higher tier reporting of current forest SOC stocks. The findings of this study can inform future research linking the variability in landscape SOC stocks to climate ranges and predicting the potential impacts of projected changes in temperature and precipitation. Given the vulnerability of mountain ecosystems to climate change, our predicted forest SOC benchmarks offer valuable insight into the future of these carbon stocks. In conclusion, this study advances our understanding of the spatial variability of forest SOC stocks along the elevational gradients of the Central Himalayas and provides a crucial baseline for evaluating the potential impacts of a changing climate on these critical carbon reservoirs.

## Supplementary Information

Supplementary Information.

## Data Availability

The predicted forest soil organic carbon (SOC) stocks covering Nepal, accompanied by the corresponding prediction uncertainty raster can be accessed at: https://doi.org/10.6084/m9.figshare.22140233^116^. The spatial layers are in GeoTIFF format with a spatial resolution of 30 m and ESPG:32644 spatial reference system.
