# Supplementary material for: Mapping soil organic carbon stocks in Nepal’s forests
Source: Sci Rep. 2023 May 19;13:8090. doi: 10.1038/s41598-023-34247-z (PMC10199042; doi:10.1038/s41598-023-34247-z)
Supplement: Supplementary file 1 — Supplementary Information. [file 41598_2023_34247_MOESM1_ESM.pdf]

Supplementary material for Quantification of soil organic carbon stocks in Nepal's forests

S0.1 Spatial predictors used in modelling

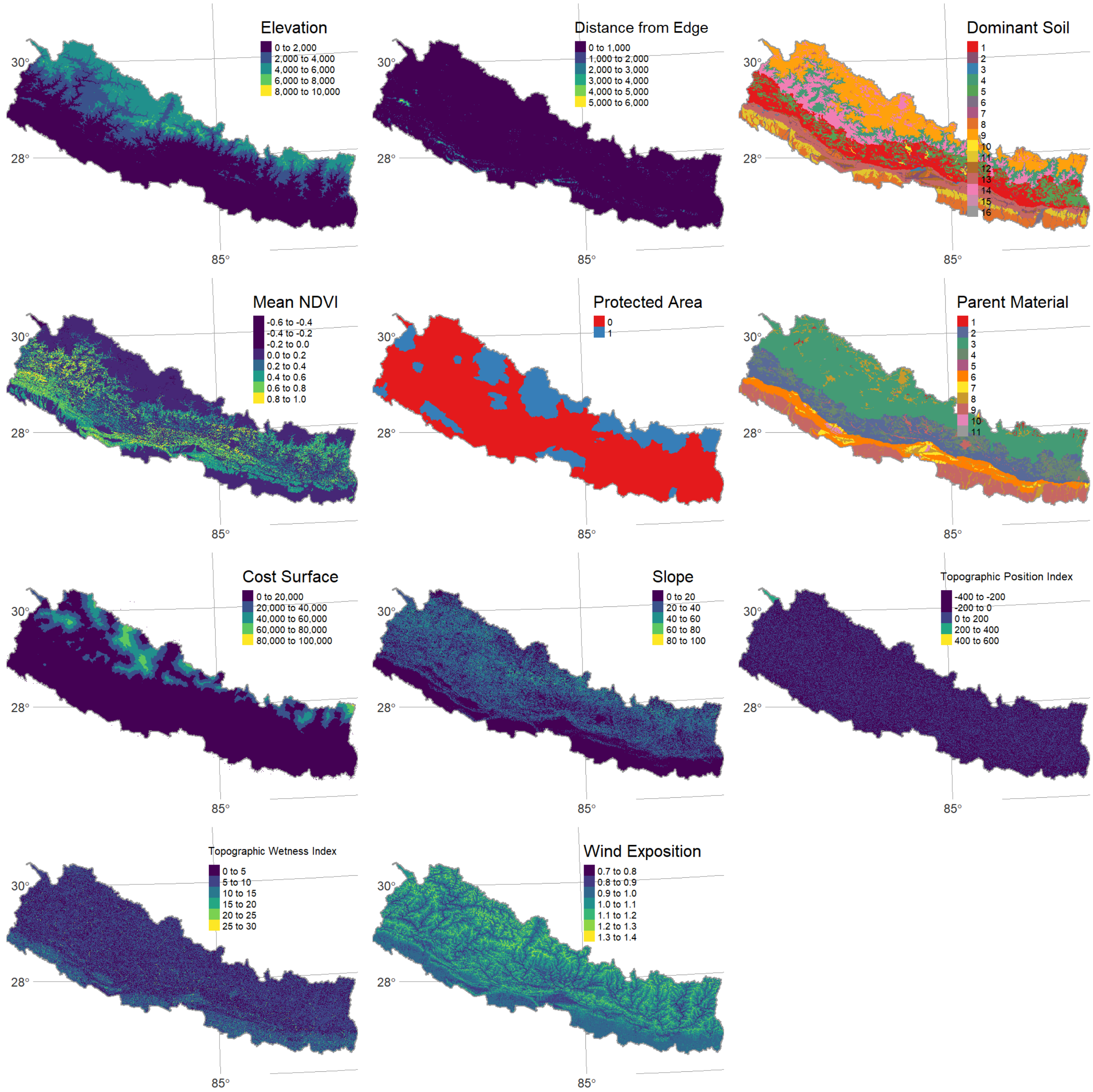

Figure S1: Predictors used for forest SOC modelling and prediction.

S0.2 Uncertainty of predicted forest SOC

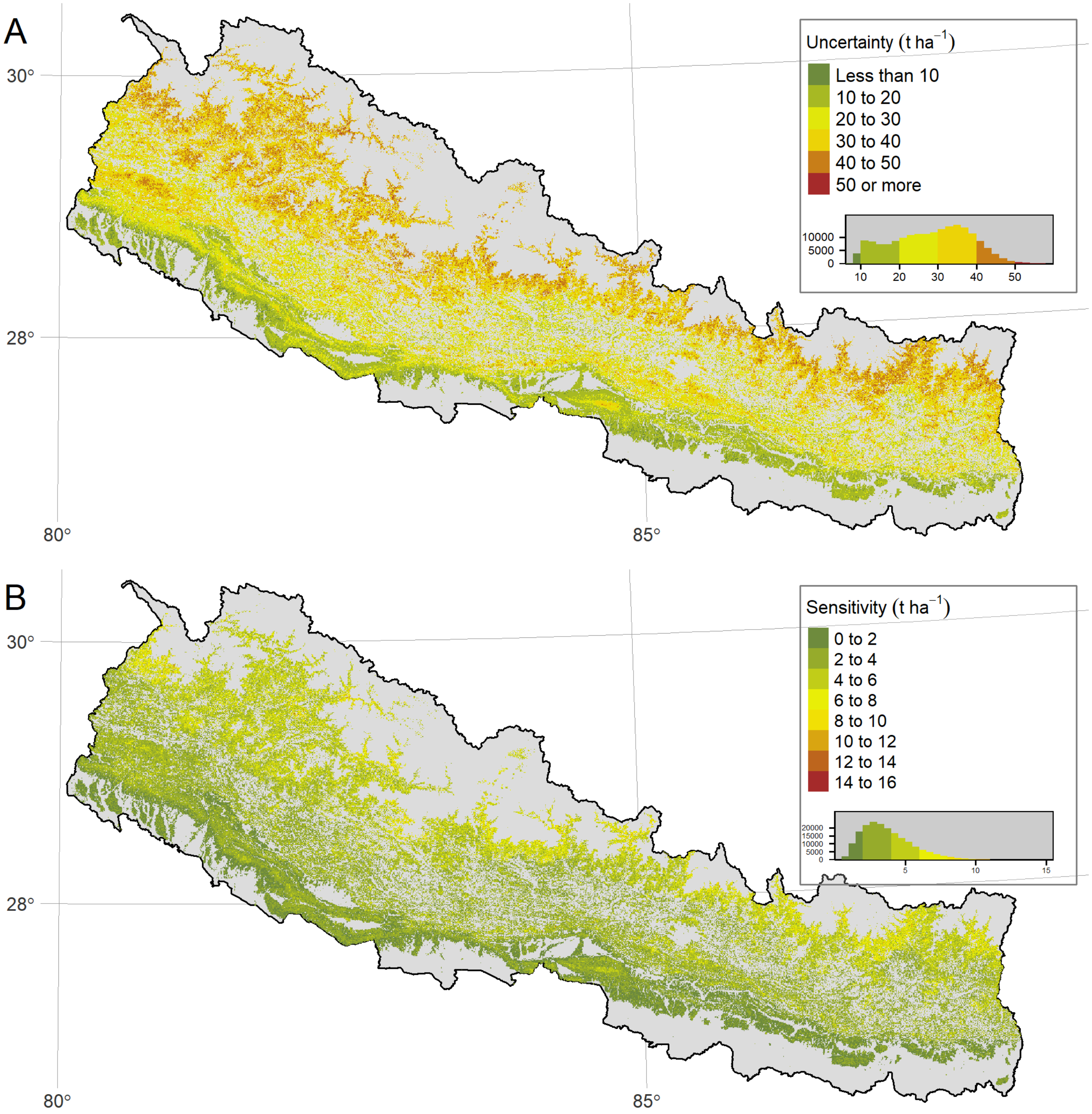

Figure S2: Standard deviation of 10-fold CV prediction of SOC. Panel A shows the model uncertainty, and Panel B shows the sensitivity due to the sample plot design.

S0.3 Map of global SOC prodcts

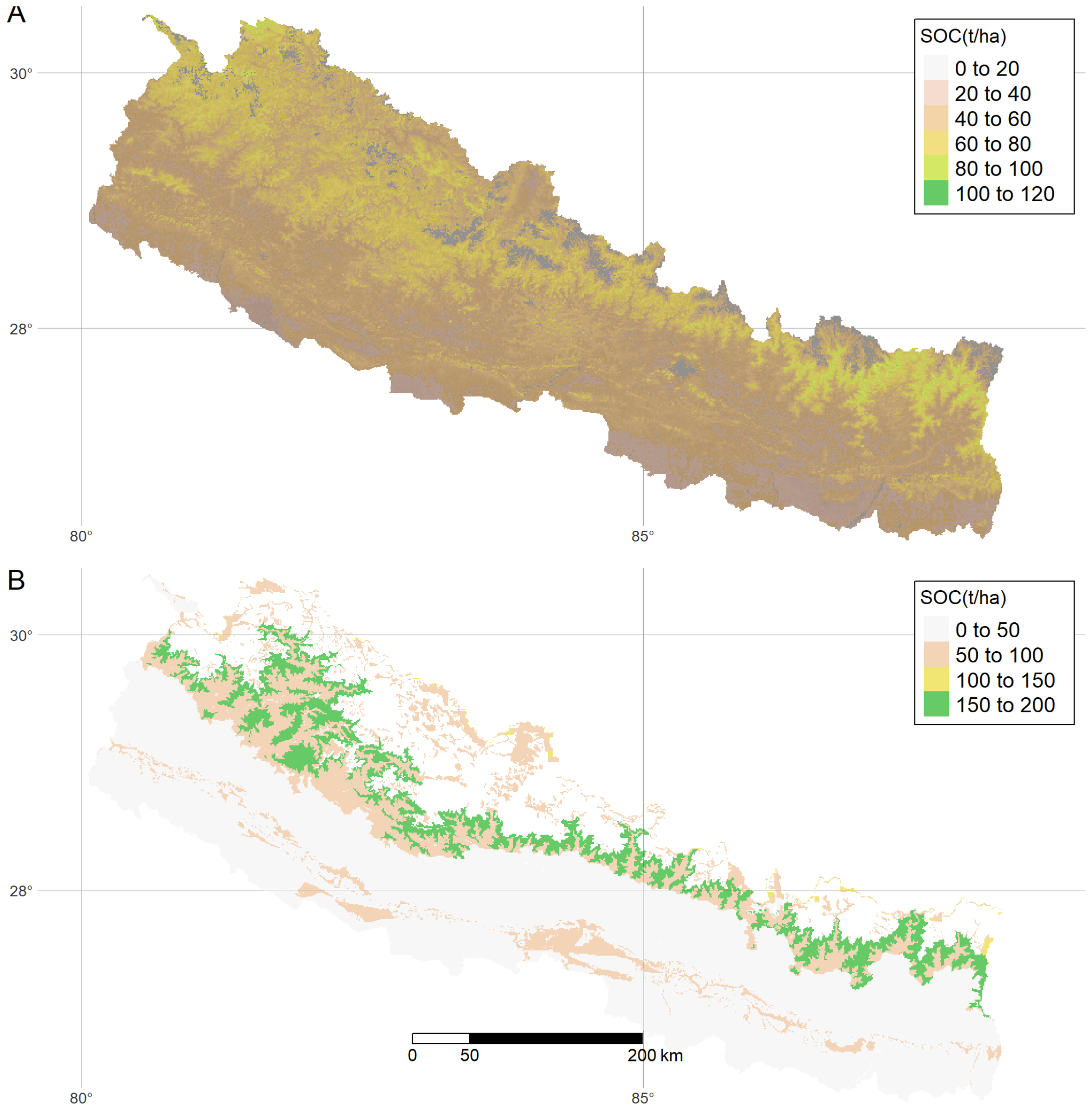

Figure S3: Global SOC map clipped with Nepal boundary. Panel A shows the GSOCmap[1], while Panel B shows SoilGrids250m 2.0[2].

S0.4 Comparison of observed forest SOC and existing global SOC data

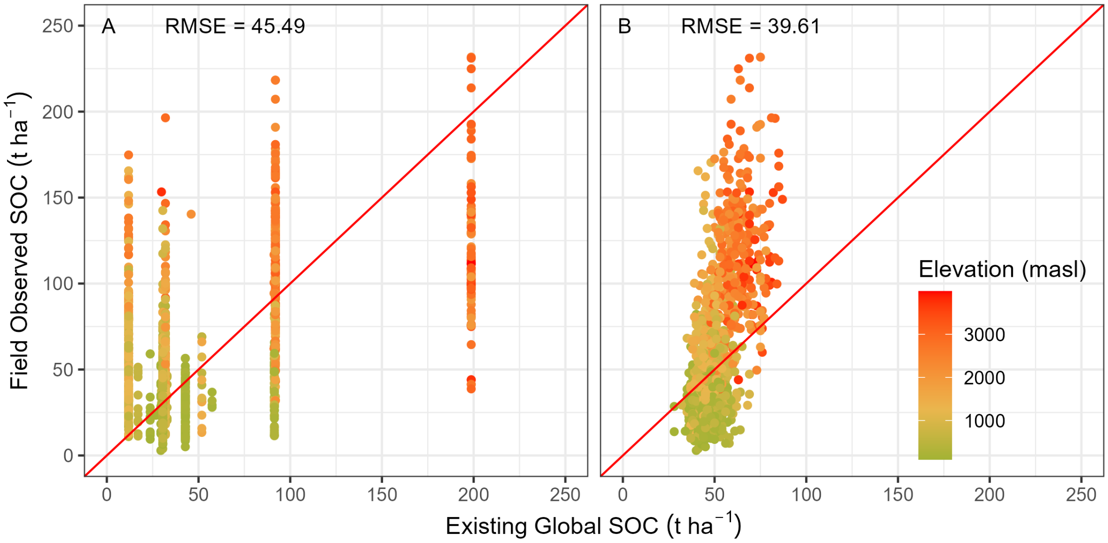

Figure S4: Comparison of field observations of forest soil organic carbon (SOC) against existing global-scale products. The color gradient represents the elevation of the input plot location. Panel A compares plot-level SOC estimates against the GSOCmap[1], while Panel B compares them against SoilGrids250m 2.0[2]. The red line in both panels represents the 1:1 line, and the root mean square error (RMSE) is expressed in t ha^{-1}.

S0.5 Comparison of observed SOC and global SOC data by elevation class

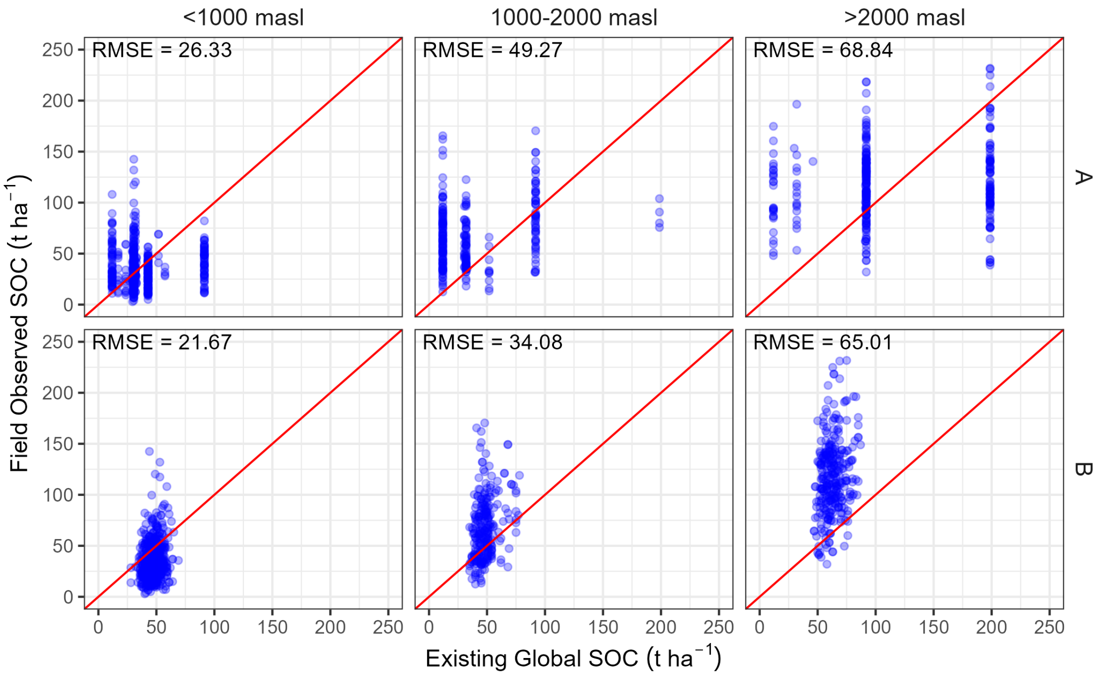

Figure S5: Comparison of field observations of forest soil organic carbon (SOC) from forest inventory plots and existing global-scale estimates. Panels in row A compare plot-level SOC estimates against GSOCmap[1] and B against SoilGrids250m[2] for three elevation ranges. The red line in all panels represents the 1:1 line, and the root mean square error (RMSE) is expressed in t ha^{-1}.

S0.6 Comparison of predicted forest SOC and existing global SOC data by elevation class

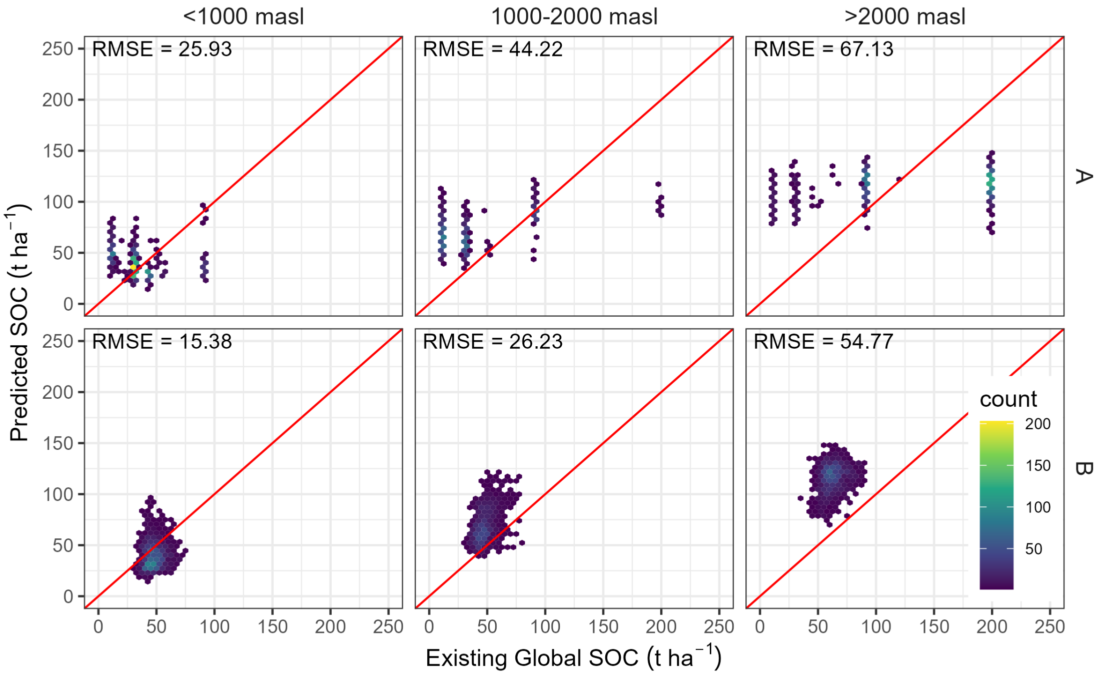

Figure S6: Comparison of predicted forest SOC in Nepal and estimates from existing global data products. Panels in row A compare predicted SOC against GSOCmap[1] and B against SoilGrids250m[2] for three elevation ranges. Random 800 points were generated for this comparison. The red line in all panels represents the 1:1 line, and the root mean square error (RMSE) is expressed in t ha^{-1}.

S0.7 Partial dependence plot for random forest model

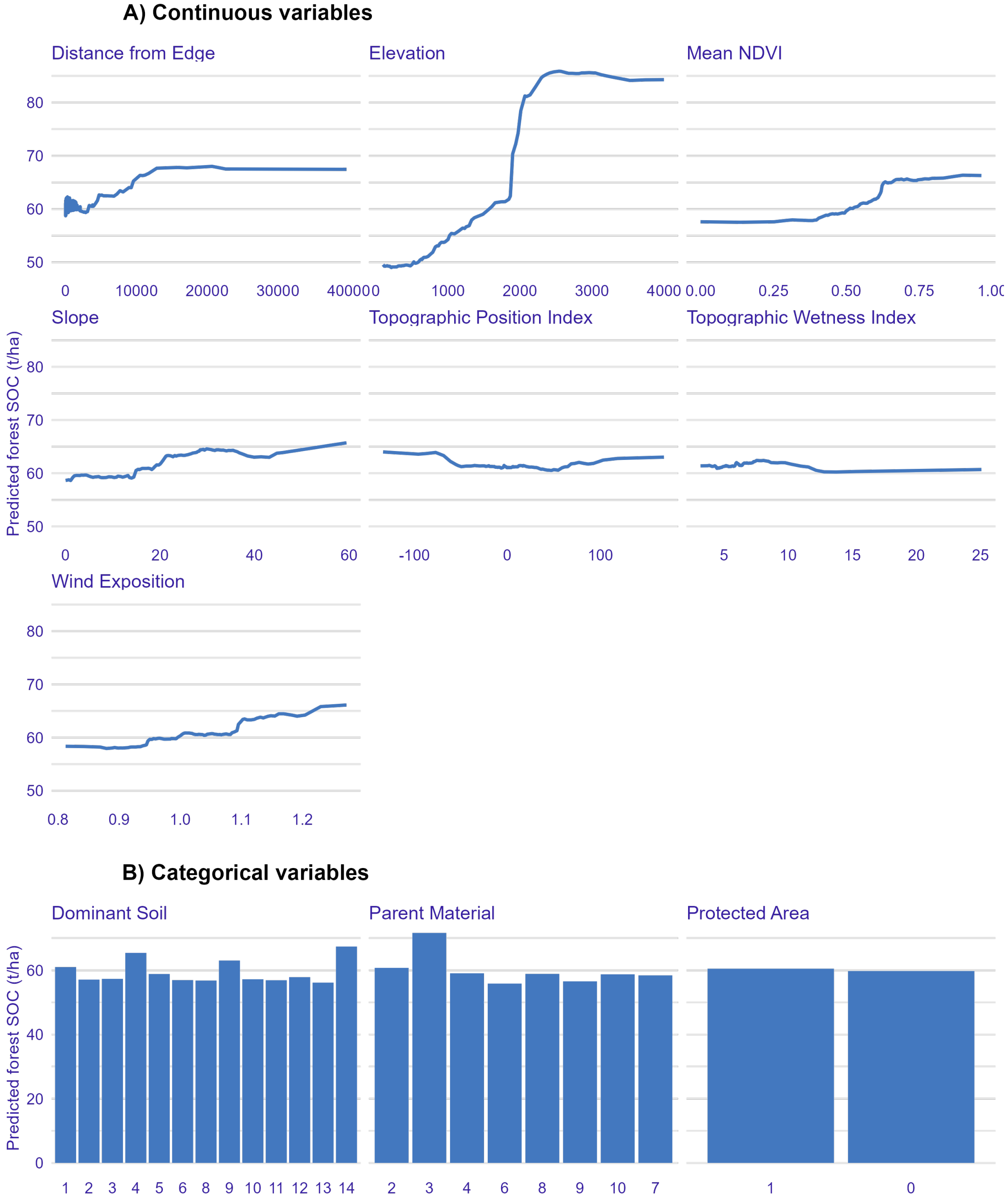

Figure S7: Partial dependence plot showing the average effect on predicted SOC as the value of covariates changes. Panel A shows the plots for continuous covariates while B shows for categorical covariates.

References

1. FAO & ITPS. *Global soil organic carbon map (GSOCmap), version 1.5.0.* http://54.229.242.119/GSOCmap/# (2018).

2. Hengl, T. *et al.* SoilGrids250m: Global gridded soil information based on machine learning. *PLOS ONE* **12**, e0169748 (2017).
